# Examining effects of rhizobacteria in relieving abiotic crop stresses using carbon‐11 radiotracing

**DOI:** 10.1111/ppl.13675

**Published:** 2022-03-29

**Authors:** Avery Powell, Stacy L. Wilder, Alexandra B. Housh, Stephanie Scott, Mary Benoit, Garren Powell, Spenser Waller, James M. Guthrie, Michael J. Schueller, Richard A. Ferrieri

**Affiliations:** ^1^ Missouri Research Reactor Center University of Missouri Columbia Missouri USA; ^2^ School of Natural Resources University of Missouri Columbia Missouri USA; ^3^ Chemistry Department University of Missouri Columbia Missouri USA; ^4^ Interdisciplinary Plant Group University of Missouri Columbia Missouri USA; ^5^ Department of Biochemistry University of Missouri Columbia Missouri USA; ^6^ Division of Plant Sciences and Technology University of Missouri Columbia Missouri USA

## Abstract

In agriculture, plant growth promoting bacteria (PGPB) are increasingly used for reducing environmental stress‐related crop losses through mutualistic actions of these microorganisms, activating physiological and biochemical responses, building tolerances within their hosts. Here we report the use of radioactive carbon‐11 (*t*_½_ 20.4 min) to examine the metabolic and physiological responses of *Zea mays* to *Azospirillum brasilense* (HM053) inoculation while plants were subjected to salinity and low nitrogen stresses. Host metabolism of “new” carbon resources (as ^11^C) and physiology including [^11^C]‐photosynthate translocation were measured in response to imposed growth conditions. Salinity stress caused shortened, dense root growth with a 6‐fold increase in foliar [^11^C]‐raffinose, a potent osmolyte. ICP‐MS analyses revealed increased foliar Na^+^ levels at the expense of K^+^. HM053 inoculation relieved these effects, reinstating normal root growth, lowering [^11^C]‐raffinose levels while increasing [^11^C]‐sucrose and its translocation to the roots. Na^+^ levels remained elevated with inoculation, but K^+^ levels were boosted slightly. Low nitrogen stress yielded longer roots possessing high levels of anthocyanins. Metabolic analysis revealed significant shifts in “new” carbon partitioning into the amino acid pool under low nitrogen stress, with significant increases in foliar [^11^C]‐glutamate, [^11^C]‐aspartate, and [^11^C]‐asparagine, a noted osmoprotectant. ^11^CO_2_ fixation and [^11^C]‐photosynthate translocation also decreased, limiting carbon supply to roots. However, starch levels in roots were reduced under nitrogen limitation, suggesting that carbon repartitioning could be a compensatory action to support root growth. Finally, inoculation with HM053 re‐instated normal root growth, reduced anthocyanin, boosted root starch, and returned ^11^C‐allocation levels back to those of unstressed plants.

## 1 INTRODUCTION

Plants encounter a variety of stresses at every developmental stage of their life, including temperature extremes, drought, flooding, salinity, and soil nutrient limitation due to pH shifts (Sharma et al., 2012). Today, approximately 30% of global crop production is lost as a result of abiotic stresses (Goswami et al., 2016). Salinity is considered one of the most critical abiotic stresses, impacting agricultural productivity from reductions in photosynthesis, respiration, and protein synthesis (Ahmad & Prasad, 2012; Dwivedi et al., 2015; Ilangumaran & Smith, 2017; Zhao et al., 2021). Salinity is a growing problem, as salinized land is expected to increase as climate change conditions can promote salt accumulation (Zhu, 2002; Corwin, 2020). Soil salinity can result in nutritional disturbances affecting plant growth due to Na^+^ ion impacts on plant–water relations (Hasegawa et al., 2000; Munns & Tester, 2008; Zahedi et al., 2012). Plants have evolved certain adaptive mechanisms that enable them to survive salinity's adverse effects. Such adaptations included accumulation of amino acids, sugars, and counterions like K^+^ that can act as potent osmolytes aiding in maintenance of cellular water status (Nuccio et al., 1999). Often, though, these natural adaptions are not sufficient to address acute stress.

Nitrogen (N) is also recognized as a major limiting nutrient for maize production (Bennett et al., 1989; Hernandez et al., 2015; Li et al., 2020; Raun & Johnson, 1999). Soil N concentrations will vary during the growing season due to surface run‐off, rainwater leaching, volatilization, and microbial consumption. Plant adaptation to limited N conditions is an important survival strategy, enabling them to successfully complete their life cycle. Typically, a low N supply leads to decreased root growth with suppression of lateral root emergence, an increased C/N ratio within tissues which causes reduced photosynthesis, and early leaf senescence (Malamy, 2005; Malamy & Ryan, 2001; Martin et al., 2002; Paul & Driscoll, 1997; Wingler et al., 2006; Q. Zhang, 2007; H. Zhang & Forde, 2000). However, a closer examination of the range of supplied N as nitrate reveals different growth morphological traits that are dependent on the level supplied. For example, growth conditions extending down from normal N levels of 10 mM nitrate to mildly stress‐inducing levels of 1 mM nitrate will cause root biomass in maize to increase (Guo, 2004), but below this level root biomass is reduced. More recently (Sun et al., 2020), it was shown that increased root biomass under mildly stress‐inducing N levels was attributable to increased root length. This root response might be considered a positive foraging strategy where longer roots are presumed better adapted to capture what N is available in the soil.

Plant growth promoting bacteria (PGPB) are finding increased use in agriculture to relieve plant stresses caused by harsh growth conditions. These organisms can activate physiological and biochemical responses within their host for mutual benefit to build natural tolerances to environmental stresses such as those caused by excess salt (Ashraf et al., 2004; Barassi et al., 2006; Dardanelli et al., 2008; Hamaoui et al., 2001; Hamdia et al., 2004; Mayak et al., 2004; Nadeem et al., 2007; Saravanakumar & Samiyappan, 2007). Often their use in agriculture has reduced losses in the field (Backer et al., 2018; Kim et al., 2012; Pankievicz et al., 2015; Pérez‐Montaño et al., 2014; Sarma et al., 2012; Yang et al., 2009).

Several PGPB commonly found in the plant rhizospheres will establish close associations with roots. Indeed, PGPB reach quite high numbers, often reaching 10^8^ colony forming units per gram fresh weight of tissue without generating noticeable host defense responses (do Amaral et al., 2017; Reinhold‐Hurek & Hurek, 1998, 2011). PGPB can alter the host plant's metabolic and physiological state through many actions (Jin et al., 2014; Kloepper, 1993; Kuan et al., 2016; van Loon, 2007) including their ability to produce phytohormones, reduce plant ethylene levels, emit volatile organic compounds that can protect plants (Cappellari & Banchio, 2020; Ryu et al., 2004; Santoro et al., 2016; Wenke et al., 2012), and enhance plant nutrient availability by releasing phosphates and micronutrients from insoluble soil sources (Housh, Powell, et al., 2021a; Housh, Benoit, et al., 2021b) and fixing non‐symbiotic nitrogen (Dobereiner & Pedrosa, 1987; Pankievicz et al., 2015; van Loon, 2007). Yet, while all these actions have been identified as contributing to the overall growth benefits seen in plants, we still know little of the mechanisms underpinning these actions.

Since PGPB are seen to be useful in modern agriculture as a viable strategy for reducing stress‐related crop losses, we applied [^11^C]‐radiotracing to a relevant crop model, *Zea mays*, inoculated with a microbial agent to map changes in the physiological and biochemical responses. Among PGPB, the genus *Azospirillum*—with an emphasis on *A. brasilense*—is probably the most studied microorganism for the mitigation of plant stress, especially salinity stress (Barassi et al., 2006; Carrozzi et al., 2012; Creus et al., 2004; Fasciglione et al., 2015; Rodríguez‐Salazar et al., 2009). Our recent success in demonstrating plant growth promotion using HM053, a functional mutant strain of *A. brasilense* in grasses (Housh, Powell, et al., 2021; Housh, Benoit, et al., 2021; Pankievicz et al., 2015; Scott et al., 2020) led us to examine its performance in relieving two very different abiotic stresses, salinity and nitrogen limitation and report on the physiological and metabolic responses of the host plant using [^11^C]‐radiotracing.

## 2 MATERIALS AND METHODS

### 2.1 Plant growth

Maize kernels from Elk Mound Seed Co. (Hybrid 8100) were dark germinated at room temperature for two days on sterilized paper towels, wetted with sterile water in a petri‐dish. Seeds were inoculated with bacterial culture as appropriate and transplanted to a growth pouch wetted with sterile Hoagland's basal salt solution for approximately one week. They were then transferred to eight‐inch plastic cones filled with Turface™ (expanded clay matrix) where the bottom portion of the cone was immersed in deionized water (Figure S1). Nutrients were introduced as Hoagland's solution every three days. Growth conditions for both set‐ups consisted of 12 h photoperiods, 500 μmol m^−2^ s^−1^ light intensity, and temperatures of 25°C/20°C (light/dark) with humidity at 70%–80% for two weeks.

Plants subjected to salinity stress were grown as described above, but cones were immersed in 100 mM NaCl. Every third day 50 ml of Hoagland's nutrient solution (1.62 g l^−1^: PhytoTechnology Laboratories) was administered to the Turface™ along with 15 ml of 100 mM NaCl. After these additions, the saline immersion solution was replaced so as not to dilute the concentration of the salt.

Plants grown under mild nitrogen limitation were administered Hoagland's nutrient solution every third day. However, this nutrient solution was prepared using a commercially available Hoagland's Basal salt (1.62 g l^−1^: Caisson Labs) that did not contain nitrogen but maintained nutrient potassium levels with KH_2_PO_4_ (monobasic) and KCl as per the company's formulation fact sheet. This nutrient solution was then enriched with the addition of KNO_3_ to achieve a level of 1 mM nitrogen compared with the 10 mM normal nitrogen nutrient solution.

### 2.2 Bacterial growth and root inoculation

HM053, a functional mutant of *A. brasilense* was obtained via a material transfer agreement between the corresponding author's institution and the Federal University of Paraná (UFPR, Curitiba, PR CEP 81531–980, Brazil). HM053 is a *Nif* ^+^ constitutively expressed strain that hyper‐fixes N_2_ excreting excess nitrogen as ammonium (Pankievicz et al., 2015). This mutant also produces high levels of auxin (Housh, Powell, et al., 2021a). HM053 originated as a natural mutant of the wild‐type strain of *A. brasilense* FP2 (Sp7 ATCC 29145 Nif^+^ Sm^r^ Nal^r^) screened through its resistance to ethylenediamine (EDA^r^; Machado et al., 1991; Pankievicz et al., 2015; Santos et al., 2017). We grew this strain in liquid NFbHP‐lactate medium following published procedures (Pankievicz et al., 2015). Cultures were washed with sterile water and diluted to approximately 10^6^–10^8^ colony forming units per milliliter of culture (CFU ml^−1^). Root inoculation involved adding 1 ml of the inoculum to a petri dish of 10–20 pre‐germinated maize seedlings and mixing in the shaking incubator for 2 h. Seedlings were placed into plastic seed germination pouches (PhytoAB Inc.) for five days before transplanting to Turface™.

### 2.3 Measuring root growth traits

Roots subjected to the described growth conditions were harvested from the Turface™, photographed using a DSLR camera and then weighed for fresh mass of tissue. Additionally, isolated roots were removed from the total biomass, suspended in a tray of water and re‐photographed. By suspending the root in water, this allowed the lateral roots to separate. Root photographs were processed using AmScope v4.11.18421 software (AmScope Inc.) to determine the average length of non‐lateral roots including primary, seminal and any crown roots, as well as the number and average length of lateral roots that were affixed to these roots.

### 2.4 Production and administration of radioactive ^11^CO_2_

^11^CO_2_ (*t*_½_ = 20.4 min) was produced on the GE 800 Series PETtrace Cyclotron located at the Missouri Research Reactor Center using high‐pressure research grade N_2_ gas target irradiated with a 16.4 MeV proton beam to generate ^11^C via the ^14^N(p,α)^11^C nuclear transformation (Ferrieri, 2003; Ferrieri & Wolf, 1983). The ^11^CO_2_ was trapped on a molecular sieve, desorbed, and quickly released into an air stream at 200 ml min^−1^ as a discrete pulse for labeling a leaf affixed within a 5 × 10 cm lighted (560 μmol m^−2^ s^−1^) leaf cell to ensure a steady level of fixation. The load leaf (source leaf two, at V2 developmental stage) affixed within the cell was pulse‐fed ^11^CO_2_ for 1 min, then chased with normal air for the duration of exposure. A PIN diode radiation detector (Carroll Ramsey Associates) attached to the bottom of the leaf cell enabled continuous measurement of radioactivity levels within the cell during the initial pulse and in the minutes directly following to give information on ^11^CO_2_ fixation and leaf export of [^11^C]‐photosynthates (Ferrieri et al., 2005).

### 2.5 Whole‐plant [ ^11^C]‐physiology measurements

After ^11^CO_2_ pulsing, plants were incubated for 1.5 h before separating the load leaf from shoots, roots, and growth media. Roots were washed in 100 ml deionized water to remove surface‐bound root exudates. Measurement of ^11^C radioactivity was performed using gamma counting and data were decay corrected to end‐of‐bombardment. The individual components (including plant material, growth material and root wash) were summed together for total plant ^11^C radioactivity and normalized based on fresh tissue weight. Individual components were used to calculate leaf export, root allocation and root exudation fractions. A 10 ml aliquot of the root washings was processed through a 1 ml column packed using 200 mesh AG1 X‐8 anion exchange resin (Bio‐Rad Laboratories Inc.). Afterwards the column and breakthrough rinse were counted for radioactivity, providing a measure of the acidic and non‐acidic root exudate fractions. However, the low levels of ^11^C radioactivity coupled with the isotope's rapid rate of decay precluded more detailed analyses for individual metabolite components within these fractions.

### 2.6 [ ^11^C]‐metabolite analyses

After ^11^CO_2_ pulsing and incubation for 20 min, leaves exposed to the radiotracer were removed and subjected to metabolite analyses following published procedures (Qu et al., 2016). Tissue was flash frozen in liquid nitrogen, ground to a fine powder and extracted in methanol: water (60:40 v/v) via sonication (Branson, Bransonic 32; Sigma‐Aldrich Corp) for 2 min at 100% amplitude in Eppendorf™ tubes. After centrifugation at 23 897 *g*  for 2 min the insoluble and soluble portions were separated. The soluble extract supernatant was placed in a separate Eppendorf™. A 20 μl aliquot of the soluble extract and the entire insoluble pellet were counted for ^11^C radioactivity using a NaI gamma counter. The insoluble portion contained mostly cell‐wall polymers, starch, and other hydrophobic metabolites. The soluble portion contained small water‐soluble compounds, including sugars, amino acids and non‐nitrogen containing organic acids. A visual of the workflow here can be found in Figure S2. All data were decay corrected back to the end of bombardment, or end of cyclotron beam.

Total soluble [^11^C]‐sugars were measured by radio thin layer chromatography (TLC) using glass backed NH_2_‐silica HPTLC‐plates (200 μm, w/UV254) purchased from Sorbent Technologies according to published procedures (Babst et al., 2013). A mobile phase consisting of 65:20:15 acetonitrile:methanol:deionized water (v/v) was used. After development, TLC plates were imaged using autoradiography on a Typhoon 9000 imager (TyphoonTM FLA 9000, GE Healthcare) and radioactivity was quantified using ImageQuant TL 7.0 software. Total [^11^C]‐sugar content was related to the ^11^C radioactivity quantified along the sample lane of the TLC plate, and then corrected to percent total fixed ^11^CO_2_ using gamma count data from the insoluble and soluble fractions.

[^11^C]‐Amino acids were analyzed following published procedures (Qu et al., 2016) using pre‐column OPA derivatization of 100 μl of the methanol: water extract (1:1 ratio) and quantified by gradient radio HPLC (Sonntek Inc.). The method used a Phenomenex Gemini 5 μm C18 (150 mm × 4.6 mm inner diameter) column heated to 30°C and mobile phase system comprised of Solvent A (95:5 0.5 M sodium acetate: methanol) and Solvent B (70:30 methanol:18MΩ water) starting at 75:25 and switching to 25:75 within 30 min at a flow rate of 0.7 ml min^−1^. On‐line fluorescence detection (340 nm excitation, 450 nm emission; Hitachi LaChrom Elite L‐2485; Sonntek Inc.) was used for correlating retention times of standards with those of [^11^C]‐amino acids in biological samples. A NaI gamma radiation detector (Ortec Inc.) enabled direct measurement of radiolabeled metabolites. Data were acquired using PeakSimple™ chromatography software (SRI Inc.) where radioactivity assigned to peaks was corrected for radioactive decay, summed for a total [^11^C]‐amino acid value and related back to the amount of ^11^C radioactivity fixed by the plant at the start of the study.

For analysis of the [^11^C]‐acidic metabolite fraction, 500 μl of leaf extract was rendered slightly basic (pH 8.5) using 1 M NaOH and the total volume was processed through a Accell QMA Plus Light Sep‐Pak™ (Waters Corporation) followed by rinsing the contents with 10 ml of DI water. Cartridges were then counted for ^11^C radioactivity on a NaI gamma counter. Metabolic contents trapped on the cartridge contained [^11^C]‐acidic metabolites including [^11^C]‐aspartic and [^11^C]‐glutamic acid. These radiolabeled amino acids were subtracted from the total [^11^C]‐acidic metabolite fraction using data obtained from their direct analysis using radio HPLC as described above. The remaining acidic metabolite fraction was not analyzed for individual metabolites.

For analysis of the [^11^C]‐basic metabolite fraction, 200 μl of tissue extract was processed through a strong cation exchange cartridge (Strata‐XL‐C, Phenomenex Inc.) followed by rinsing the cartridge using 10 ml of DI water. The cartridge was subjected to gamma counting for quantification of this metabolite pool. We did not analyze for individual radiolabeled metabolites within this fraction.

### 2.7 Inductively coupled plasma‐mass spectrometry (ICP‐MS)

For ICP‐MS analyses of Na^+^, K^+^ and Ca^2+^, roots and shoots were harvested from salt stressed plants including HM053 inoculated salt stressed plants. Tissues were dried in an oven, weighed and ground up by hand using a mortar and pestle. The samples were digested in 3.0 ml of concentrated nitric acid at 190°C using a Milestone Ethos Plus (Milestone SRL) microwave digestion system, then were diluted to 50 ml with ultrapure water followed by gravimetric dilution by a factor of 10 with 0.45 M nitric acid. Samples were analyzed via Perkin‐Elmer NexION ICP‐MS in Kinetic Energy Discrimination mode. Reference materials included NIST SRM 1570 spinach leaves and NIST SRM 1573 tomato leaves prepared as samples and analyzed in the same way. Internal standards at known concentrations were prepared from stock solutions (High Purity Standards) and used to calibrate instrument response.

### 2.8 Starch analysis

Plant tissue starch levels were analyzed using Sigma‐Aldrich Starch Assay Kit (Sigma‐Aldrich Technical Bulletin, 2019) using enzymes to break down starch to glucose and further reactions to create a colored product for absorbance measurement at 540 nm using UV–vis spectrometry (Evolution 201 UV–vis spectrophotometer, Thermo Fisher Scientific Inc.). Maize leaf (100–200 mg) tissue was ground using liquid nitrogen chilled 2 ml Eppendorf tubes in a ball mill grinder (Retsch Inc.). Root tissue (500–1000 mg) was ground using a liquid nitrogen chilled mortar and pestle. After freeze grinding, 5 ml of 80% aqueous ethanol was added to the root portions and 1 ml of this solution was added to the leaf portions. Samples were heated for 5 min at 85°C with constant agitation using a Cimarec hot plate (Thermo Fisher Scientific Inc.) equipped with an orbital shaker platform (SP Bel‐Art Inc.). The resulting solutions were centrifuged for 10 min at 1000*g*, supernatants were removed, and pellets re‐suspended in equivalent volumes of 80% aqueous ethanol before being subjected to centrifugation once more. This process removes the latent glucose residing in the plant tissues before digesting the starch to glucose.

To begin the starch digestion process, 0.2 ml of the 80% ethanol solution was added to each pellet. For each root and leaf sample, 2 ml, and 1.5 ml of deionized (DI) water were added, respectively, and then 0.02 ml of α‐Amylase to each. Each tube was vortexed, mixed, and incubated for 10 min in a boiling water bath. Samples were removed, cooled to ambient temperature, and diluted to 10 ml for roots and 2 ml for leaves using DI water. Aliquots of these diluted samples, 1 ml for root and 100 μl for leaf samples, were mixed with amyloglucosidase (50 units ml^−1^) at a 1:1 ratio (v/v) and incubated in a thermomixer (Eppendorf Inc.) at 60°C for 15 min using constant agitation. After cooling to ambient temperature, each tissue sample was again diluted in DI water (10 ml for roots, and 2 ml for leaves).

For the glucose assay, 1 ml of each sample was placed in a new falcon tube. Two milliliters of *o*‐dianisidine, 0.1 mg ml^−1^, and reconstituted glucose oxidase/peroxidase, 12.5 units ml^−1^ in water was added to the sample, mixed, and placed on the hot plate. Samples were incubated for 30 min at 37°C with constant agitation. The enzymatic reaction was quenched by adding 2 ml of 6 M sulfuric acid. The final extracts were measured using a UV Spectrophotometer at 540 nm (Thermo Scientific) and the amount of glucose derived from starch breakdown was reported as mg gfw^−1^ tissue.

### 2.9 Anthocyanin analysis

Approximately 1 g of fresh frozen maize roots was hand ground using a mortar and pestle. The frozen ground sample was weighed and mixed in a 15 ml Falcon tube with 2 ml 2 M HCl. The tube was capped and heated for 1 h at 50°C in a water bath. Afterwards, the contents were centrifuged, and the supernatant analyzed on a UV Spectrophotometer at 520 nm (Thermo Scientific) for presence of anthocyanins. Instrument response was calibrated against authentic anthocyanin standards. Root anthocyanin levels were reported as nmol of anthocyanin per gram fresh weight of tissue (nmol gfw^−1^).

### 2.10 Bacterial quantification—Drop plate assay

Bacterial quantifications were performed concurrently with metabolite studies. Plants were harvested from the growth media and rinsed in DI water. Two separate 1–1.5‐inch sections of roots were taken from each plant and weighed (approximately 100–300 mg total). The sample was ground with mortar and pestle in 1 ml of 1% saline. Five serial dilutions were conducted: the first with 100 μl of the ground extract into 900 μl of 1% saline and each subsequent dilution being 100 μl of the previous dilution into 900 μl of 1% saline. Each serial dilution was plated in triplicate by 10 μl drops onto agar plates fortified with lactate growth media and incubated at 3°C for 48–72 h before counting. The dilution that contained 3–40 colony forming units (CFU) per 10 μl drop was counted and used to perform calculations yielding the data plotted in Figure S3. Here the Log_10_ CFU was plotted against treatment type.

### 2.11 Statistical analysis

Data were subjected to one‐way analysis of variance (anova) in R using the SigmaPlot 14.5. Tukey's HSD test was used for post hoc correction of comparisons across the inoculated and non‐inoculated stressed plants and the unstressed non‐inoculated controls, as well as between inoculated and non‐inoculated stressed plants at a significance level of *P* < 0.05.

## 3 RESULTS

### 3.1 Root growth traits

**FIGURE 1:**
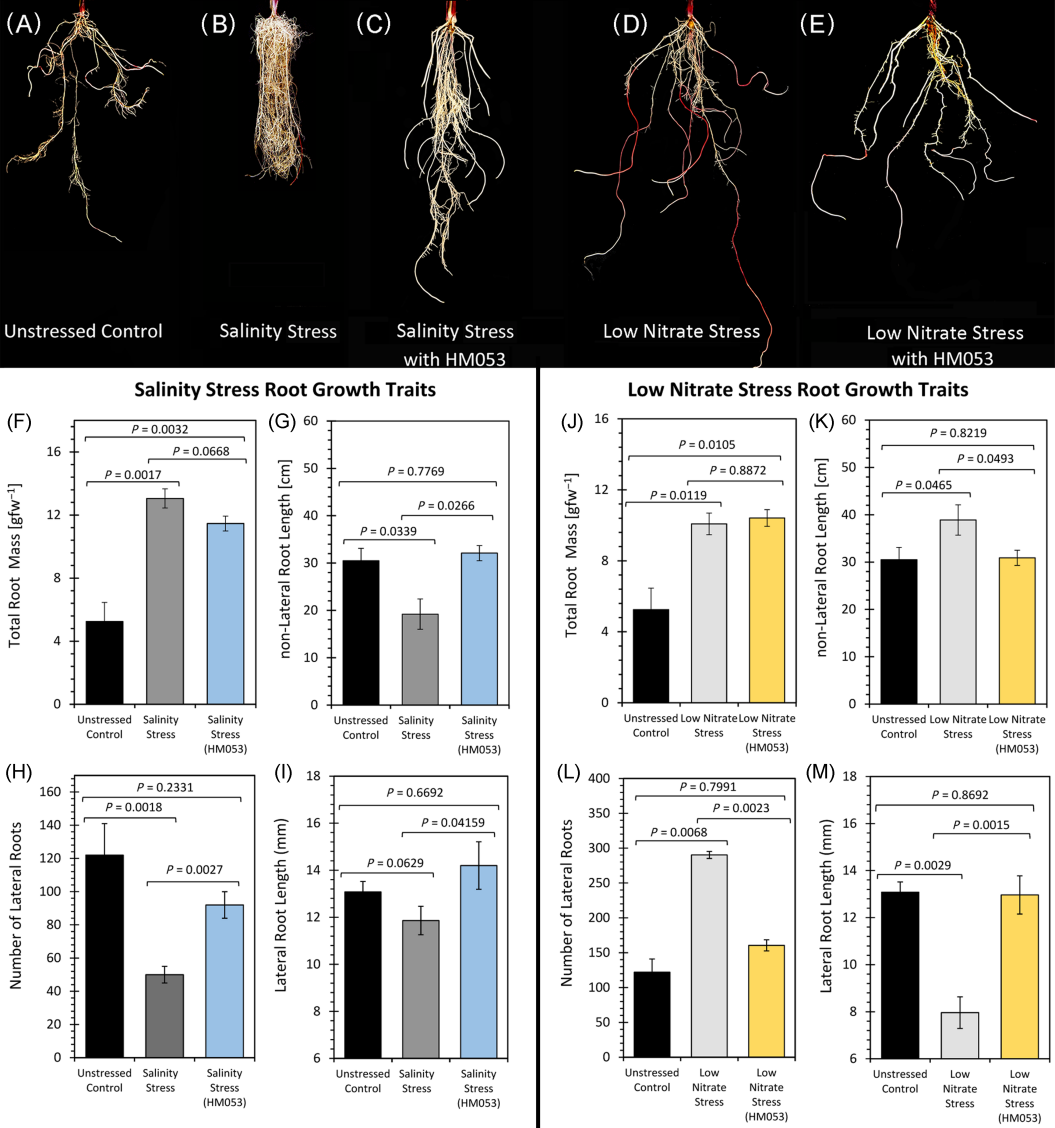
Effect of salinity and low nitrate stresses on root growth traits. (A–E) Photographs reflect the different growth traits of maize roots when plants were grown under study conditions including salinity stress and low nitrogen stress with and without inoculation using HM053 *Azospirillum brasilense*. (F and J) Total root fresh masses for salinity and low nitrogen stresses, respectively. (G and K) Dimensions of primary, seminal and crown roots were measured by tracing isolated “floated” roots in digital photos using AmScope software. Data reflect averages of all roots sampled by this method for salinity and low nitrogen stresses, respectively. (H and L) The number of lateral roots were tallied from individual roots that were isolated from the plant and analyzed in (G) and (K). (I and M) The length of individual lateral roots was measured using the same procedures used in (G) and (K) and averaged. Data (± se) reflect *n* = 6–9 biological replicates for all measurements reported in these figures. Levels of significance are reflected by the included *P*‐values for each figure when comparing the following: (i) non‐inoculated stressed plants to unstressed control plants; (ii) inoculated stressed plants to non‐inoculated stressed plants; and (iii) inoculated stressed plants to unstressed control plants. *P*‐values less than 0.05 were considered statistically significant

Because certain abiotic stresses are known to affect root growth and root architecture, we wanted to examine root growth traits as a function of salt exposure and nitrogen limitation as well as the combination of these abiotic stresses with administration of the microbial inoculant *A. brasilense* (HM053). Results in Figure 1 show a representative sampling of maize root images from plants grown under the different experimental conditions outlined above. Chronic exposure to salt during growth (Figure 1B) resulted in overall shorter non‐lateral roots (Figure 1G) and shorter lateral roots (Figure 1I) compared to those under normal growth conditions. However, salinity stress significantly increased the number of lateral roots (Figure 1H) which contributed to an increased overall root mass (Figure 1F) relative to normal growth conditions. The introduction of HM053 *A. brasilense* inoculation to plants in this salinity stress group resulted in a re‐establishment of root growth traits of unstressed plants (Figure 1C) by causing significant increases in all root lengths (including both non‐lateral roots and lateral roots) relative to non‐inoculated stressed plants (Figure 1G,I). Drop plate assays verified that microbial growth under this stress regime was two‐to‐three orders of magnitude higher than non‐inoculated plants (Figure S3). Although total root masses were significantly reduced in inoculated relative to non‐inoculated stressed plants, root masses remained significantly elevated relative to normal growth conditions (Figure 1F).

**FIGURE 2:**
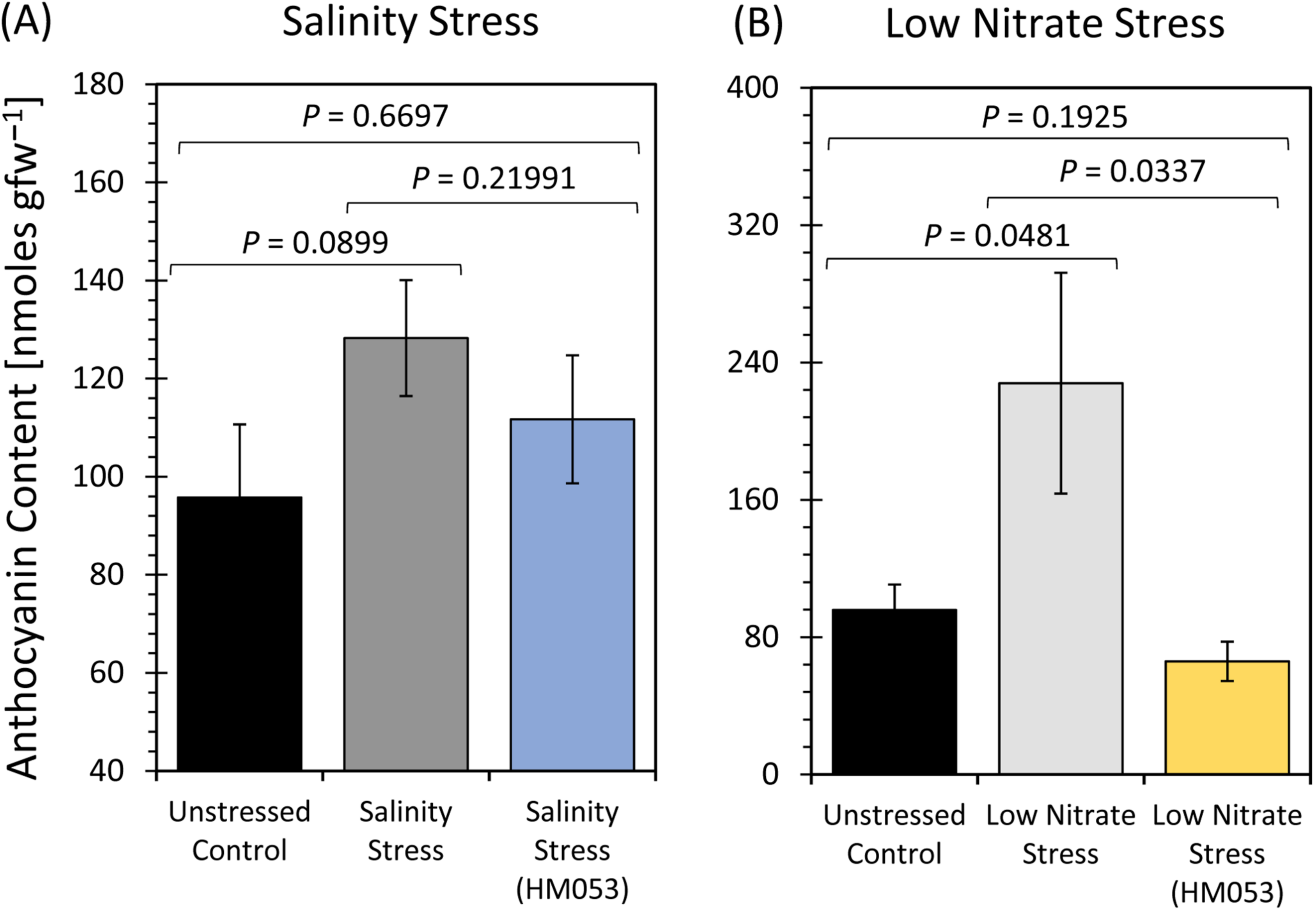
Anthocyanin analysis: Root anthocyanins were measured using UV absorption on tissue extracts. Data reflect average values (± se) from *n* = 6–9 biological replicates. (A) Represents data on salinity stress. (B) Represents data on low nitrogen stress. Levels of significance are reflected by the included *P*‐values for each figure when comparing the following: (i) non‐inoculated stressed plants to unstressed control plants; (ii) inoculated stressed plants to non‐inoculated stressed plants; and (iii) inoculated stressed plants to unstressed control plants. *P*‐values less than 0.05 were considered statistically significant

Contrary to the above observations, nitrogen limitation caused longer non‐lateral roots (Figure 1D,K), but shorter lateral roots (Figure 1M) relative to normal unstressed growth conditions. However, the number of lateral roots was significantly elevated under nitrogen limiting growth (Figure 1L) relative to unstressed growth conditions. The overall effect was a significantly elevated total root mass (Figure 1J). It was noted that roots also exhibited a strong red pigmentation when grown under nitrogen limiting conditions. Upon closer examination, we found that low nitrogen stress caused a significant elevation in root anthocyanin levels (Figure 2) from 96 ± 11 nmol gfw^−1^ in unstressed control plants to 230 ± 30 nmol gfw^−1^. It was also noted that salinity stress did not appear to affect root anthocyanin levels. HM053 *A. brasilense* inoculation of plants grown under nitrogen limitation resulted in significant reductions in non‐lateral root lengths (Figure 1K) like those of unstressed control plants. Similar behavior was seen in the growth trait of lateral roots (Figure 1L,M). That is, lateral root growth increased in length and decreased in number replicating growth traits of unstressed control plants. Again, drop plate assays verified that microbial growth under this low N stress regime was significantly higher than non‐inoculated plants by several orders of magnitude (Figure S3). We noted that the red root pigmentation in inoculated plants was less pronounced relative to non‐inoculated nitrogen stressed plants (Figure 1E) which, upon closer examination, it was noted that root anthocyanin levels were significantly lower (68 ± 12 nmol gfw^−1^) than the non‐inoculated stressed plants (Figure 2) returning to levels like that seen for normal unstressed plants. However, total root mass remained significantly elevated (Figure 1J).

### 3.2 Plant physiological responses

**FIGURE 3:**
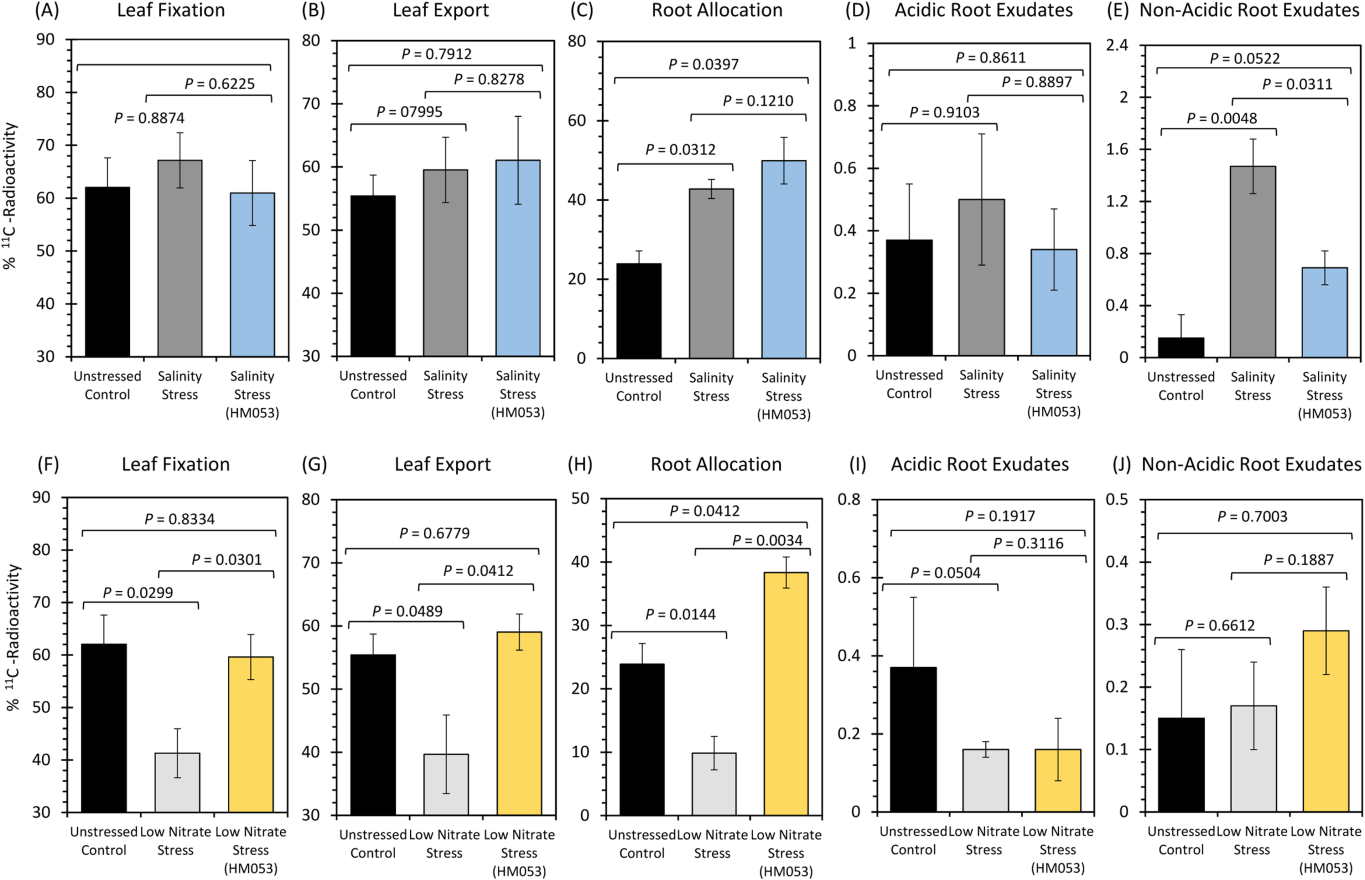
Whole‐plant physiological measurements: Carbon‐11 aids in mapping maize physiological responses to applied abiotic stresses of salinity and nitrogen limitation and to the introduction of microbial inoculant onto these stresses. (A and F) Leaf tissue normalized ^11^CO_2_ fixation values for salinity and low nitrogen stresses, respectively, where all data are presented as % ^11^C‐radioactivity in the pulse applied to the leaf cuvette. (B and G) Leaf export values of [^11^C]‐photosynthates (measured at 1.5 h post ^11^CO_2_ pulse) for salinity and low nitrogen stresses, respectively, are presented as % ^11^C‐radioactivity fixed by the plant and calculated as [(Total plant activity − Leaf activity)/Total plant activity] × 100. (C and H) Root allocation values of [^11^C]‐photosynthates for salinity and low nitrogen stresses, respectively, are presented as % ^11^C‐radioactivity fixed by the plant and calculated as [Root activity/Total plant activity] × 100. (D and I) Root exudation values of acidic [^11^C]‐substrates measured at 1.5 h post ^11^CO_2_ pulse for salinity and low nitrogen stresses, respectively, are presented as % ^11^C‐radioactivity fixed by the plant. (E and J) Root exudation values of non‐acidic [^11^C]‐substrates measured at 1.5 h post ^11^CO_2_ pulse for salinity and low nitrogen stresses, respectively, are presented as % ^11^C‐radioactivity fixed by the plant. For all figures graphical bars reflect average values ±se for *n* = 6–9 biological replicates. Levels of significance are reflected by the included *P*‐values for each figure when comparing the following: (i) non‐inoculated stressed plants to unstressed control plants; (ii) inoculated stressed plants to non‐inoculated stressed plants; and (iii) inoculated stressed plants to unstressed control plants. *P*‐values less than 0.05 were considered statistically significant

Using carbon‐11 administered to plants as ^11^CO_2_, the following physiological traits were measured for the above growth conditions: (i) leaf fixation (Figure 3A,F); (ii) leaf export of [^11^C]‐photosynthates (Figure 3B,G); (iii) allocation of [^11^C]‐photosynthates to root tissues (Figure 3C,H); (iv) exudation of acidic [^11^C]‐substrates (Figure 3D,I); and (v) exudation of non‐acidic [^11^C]‐substrates (Figure 3E,J). For leaf fixation, data were presented as percent of ^11^C radioactivity in the pulse. In all other cases, data were presented as a percent of the ^11^C radioactivity fixed by the plant. Levels of significance are shown in these figures by the representative *P*‐values. Comparisons were made between non‐inoculated stressed and unstressed control plants, HM053 inoculated stressed and non‐inoculated stressed plants, and HM053 inoculated stressed plants and non‐inoculated unstressed control plants. Results indicated that salinity stress had no impact on leaf ^11^CO_2_ fixation (67 ± 6%) relative to non‐inoculated unstressed plants (62 ± 6%). The administration of the microbial inoculant onto this stress did not change this behavior (61 ± 6%). Contrary to this, nitrogen limitation significantly decreased ^11^CO_2_ fixation (41 ± 4%) relative to non‐inoculated unstressed plants, while introduction of the microbial inoculant under nitrogen limitation significantly increased ^11^CO_2_ fixation (60 ± 5%) relative to non‐inoculated plants grown under the same stress condition reinstating normal physiological behavior.

Similarly, salinity stress had no effect on leaf export of [^11^C]‐photosynthates (60 ± 5%) relative to non‐inoculated unstressed plants (55 ± 3%), nor did the introduction of the microbial inoculant alter this behavior (61 ± 7%). However, nitrogen limitation significantly decreased leaf export (40 ± 6%) relative to non‐inoculated unstressed plants while introduction of the microbial inoculant onto this stress significantly increased leaf export (59 ± 3%) relative to stressed non‐inoculated plants, reinstating normal unstressed physiological behavior.

Further, salinity stress significantly increased allocation of [^11^C]‐photosynthates to root tissues (43 ± 3%) relative to non‐inoculated unstressed plants (24 ± 3%). Introduction of the microbial inoculant here showed similar elevated responses (50 ± 5%) as non‐inoculated salt stressed plants. Nitrogen limitation, however, significantly decreased root allocation (10 ± 2%) relative to non‐inoculated unstressed plants while the introduction of microbial inoculant onto this stress significantly increased allocation (38 ± 3%) relative to non‐inoculated stressed plants reinstating and even surpassing normal unstressed physiological behavior.

Non‐inoculated unstressed plants exhibited 0.37 ± 0.21% of acidic exudates and 0.15 ± 0.11% of non‐acidic [^11^C]‐exudates. Salinity stress did not influence the level of acidic [^11^C]‐exudates (0.50 ± 0.22%), however, it caused a significant upward shift in non‐acidic exudates (1.47 ± 0.09%). Introduction of the microbial inoculant onto this stress significantly reduced the non‐acidic fraction (0.69 ± 0.19%) to levels similar to unstressed control plants. No change was observed in the acidic fraction under these conditions (0.34 ± 0.15%). Low nitrogen stress significantly reduced exudation of acidic [^11^C]‐substrates (0.16 ± 0.05%) relative to non‐inoculated unstressed plants. Introduction of the microbial inoculant onto this stress did not affect the acidic fraction (0.16 ± 0.10%), nor was the non‐acidic fraction (0.29 ± 0.10%) significantly affected.

### 3.3 ^11^C‐metabolite profiling

**FIGURE 4:**
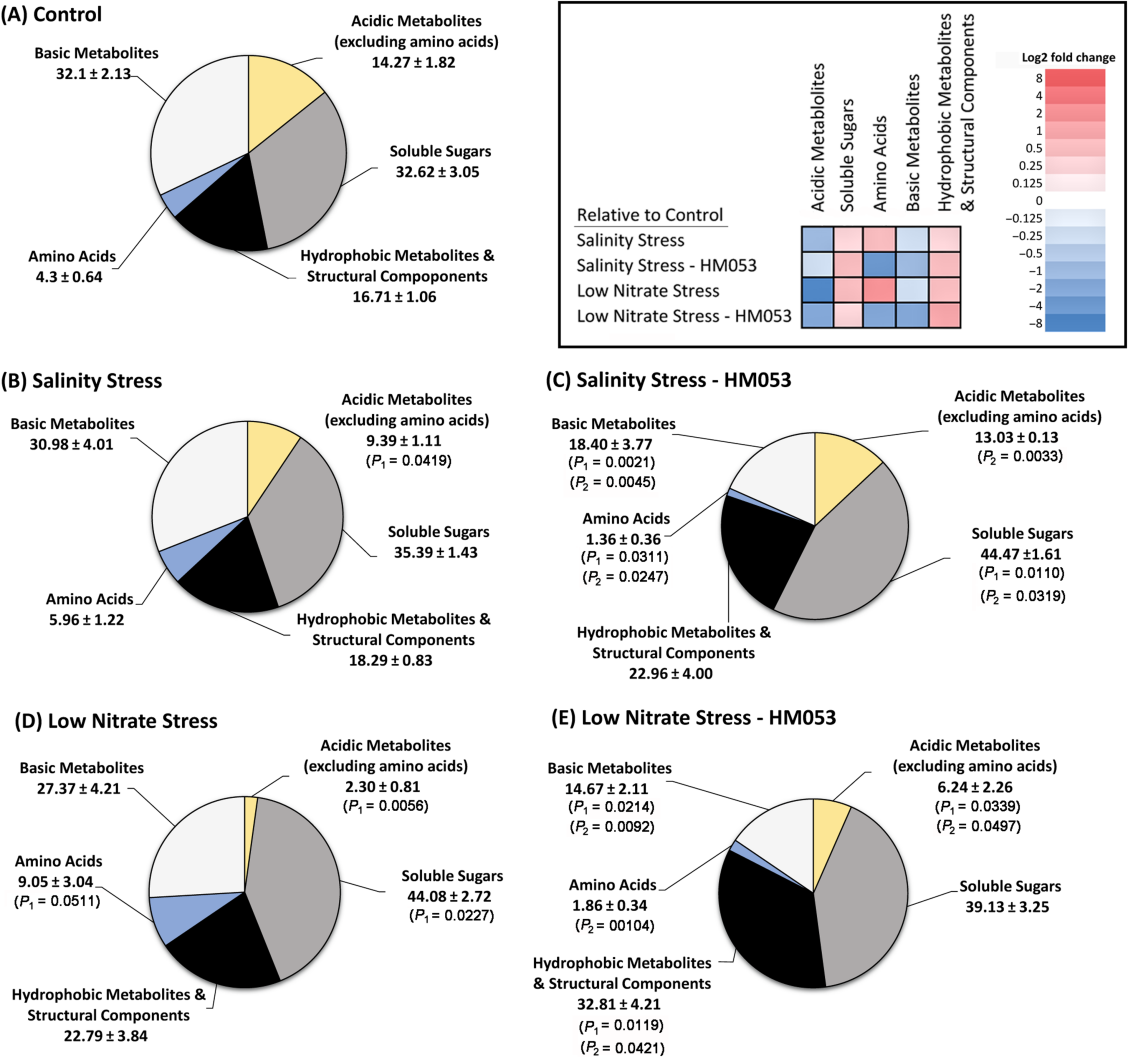
Metabolic landscape of ^11^C‐partitioning: Carbon‐11 aids in mapping changes in maize carbon metabolism as a function of applied abiotic stresses of salinity and nitrogen limitation and to the introduction of microbial inoculant onto these stresses. The metabolic landscape reflects the partitioning of “new” carbon (as ^11^C) into different metabolic pools of the load leaf tissue when harvested 20 min after the initial ^11^CO_2_ pulse. Data represent % ^11^C‐radioactivity fixed by the plant and reflect average values (± se) from *n* = 6–9 biological replicates. Metabolite pools that were examined included acidic metabolites (excluding amino acids), basic metabolites, soluble sugars, amino acids and other hydrophobic and structural pools that were not extractable in methanol:water. Levels of significance are reflected by the included *P*‐values for each sector when comparing the following: (i) non‐inoculated stressed plants to unstressed control plants (denoted by *P*_1_); (ii) inoculated stressed plants to non‐inoculated stressed plants (denoted by *P*_2_); and (iii) inoculated stressed plants to unstressed control plants (denoted by *P*_1_). *P*‐values less than 0.05 were considered statistically significant. Nonsignificant *P*‐values were not shown. For greater clarity of the systematic changes, a color‐coded heat map is presented as an inset where Log_2_‐fold changes in metabolite pools are represented from shades of blue reflecting decreased trends to shades of red reflecting increased trends

**FIGURE 5:**
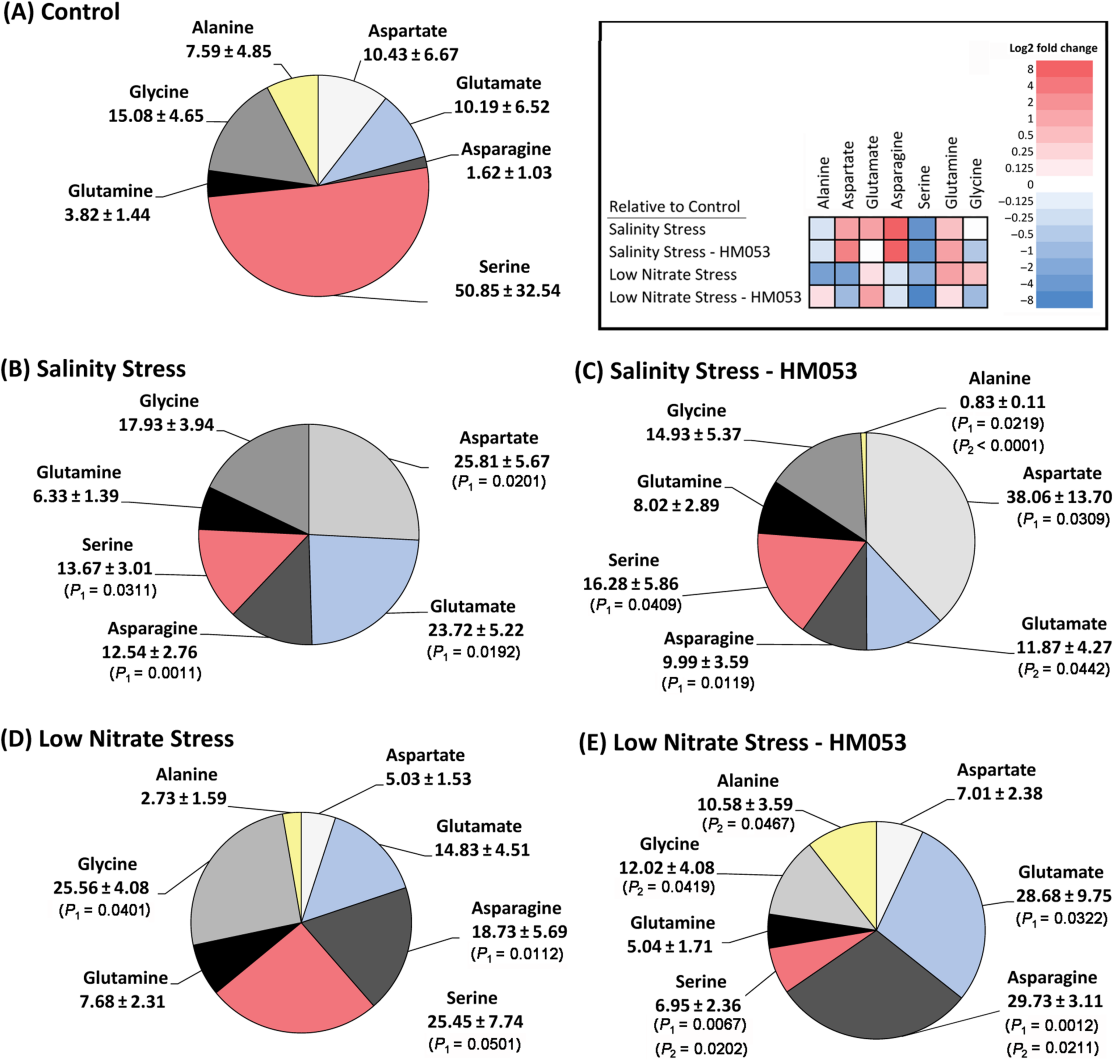
^11^C‐partitioning into individual amino acids. The ^11^C‐amino acids were broken down further into individual major amino acid pie sections in this figure where values represent an average relative distribution of the amino acid pie shown in Figure 4 (± se) from *n* = 6–9 biological replicates. Levels of significance are reflected by the included *P*‐values for each sector when comparing the following: (i) non‐inoculated stressed plants to unstressed control plants (denoted by *P*_1_); (ii) inoculated stressed plants to non‐inoculated stressed plants (denoted by *P*_2_); and (iii) inoculated stressed plants to unstressed control plants (denoted by *P*_1_). *P*‐values less than 0.05 were considered statistically significant. Nonsignificant *P*‐values were not shown. For greater clarity of the systematic changes, a color‐coded heat map is presented as an inset where Log_2_‐fold changes in metabolite pools are represented from shades of blue reflecting decreased trends to shades of red reflecting increased trends

**FIGURE 6:**
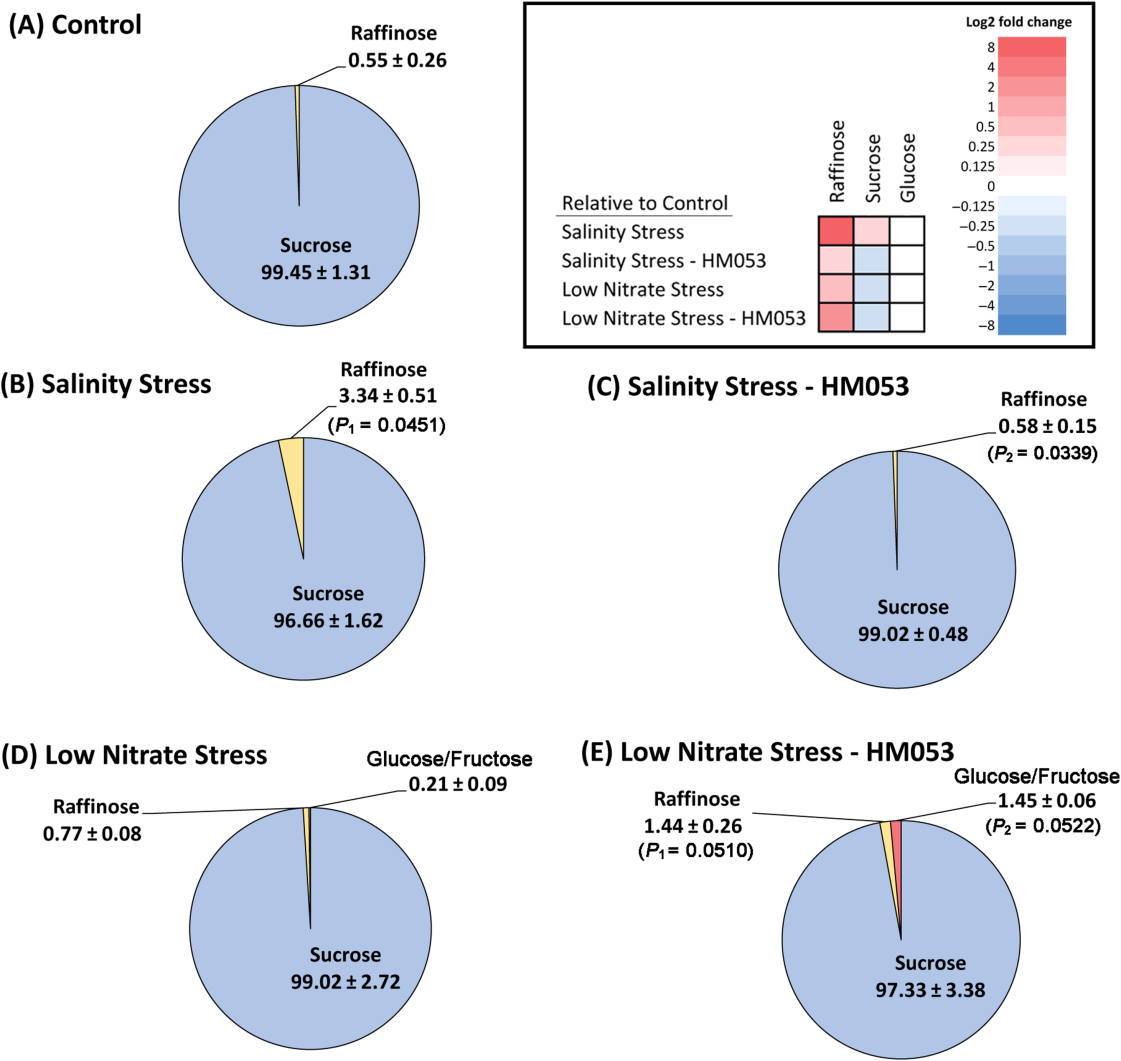
^11^C‐partitioning into individual sugars. The ^11^C‐soluble sugars were broken down further into individual sugar pie sections in this figure where values represent an average relative distribution of the soluble sugar pie shown in Figure 4 (± se) from *n* = 6–9 biological replicates. Levels of significance are reflected by the included *P*‐values for each sector when comparing the following: (i) non‐inoculated stressed plants to unstressed control plants (denoted by *P*_1_); (ii) inoculated stressed plants to non‐inoculated stressed plants (denoted by *P*_2_); and (iii) inoculated stressed plants to unstressed control plants (denoted by *P*_1_). *P*‐values less than 0.05 were considered statistically significant. Nonsignificant *P*‐values were not shown. For greater clarity of the systematic changes, a color‐coded heat map is presented as an inset where Log_2_‐fold changes in metabolite pools are represented from shades of blue reflecting decreased trends to shades of red reflecting increased trends

Often, metabolic profiling of how plants use their newly acquired carbon resources can provide insights into the state of the plant, as well as mechanisms of action when PGPB inoculants are introduced during growth to alter their host's metabolic state (Housh, Powell, et al., 2021a). Here we profiled the metabolic landscape of newly fixed ^11^CO_2_ as that carbon source was quickly partitioned between the different metabolite pools of the leaf tissue. In unstressed non‐inoculated plants (Figure 4A) partitioning of new carbon generated the following baseline profile: structural and hydrophobic metabolites (16.71 ± 1.06%); soluble sugars (32.62 ± 3.05%); amino acids (4.30 ± 0.64%); basic metabolites (32.10 ± 2.13%); acidic metabolites (14.27 ± 1.82%) excluding amino acids. Salinity stress (Figure 4B) imposed no significant change in this partitioning distribution with the exception that the acidic metabolite pool was decreased (9.39 ± 1.11%) relative to non‐inoculated unstressed plants. However, a closer inspection for changes of metabolite components within individual fractions revealed additional insight into the stress response. For example, the amino acid pool appeared unaffected by salinity stress, however, examination of the individual amino acids revealed significant increases in “new” carbon partitioning into [^11^C]‐aspartic acid, [^11^C]‐glutamic acid and [^11^C]‐asparagine, while reducing levels of [^11^C]‐serine (Figure 5A,B). Additionally, salinity stress did not alter the total pool size of the ^11^C‐soluble sugars (Figure 4A,B), contrary to prior studies that have shown that the addition of NaCl up to 500 mM significantly increased shoot levels of soluble sugars (Liu et al., 2008; G. Q. Wu et al., 2013). However, our salinity stress studies revealed a marked increase in raffinose levels within the sugar pool from 0.55 ± 0.26% in unstressed plants to 3.34 ± 0.51% in salinity stressed plants (Figure 6A,B).

Introduction of the microbial inoculant onto salinity stress (Figure 4C) resulted in a significant increase in the soluble sugar pool to 44.47 ± 1.61% compensated by a significant decrease in the amino acid pool to 1.36 ± 0.44% and basic metabolite pool to 18.40 ± 3.77% relative to both non‐inoculated stressed and unstressed plants. The acidic metabolite pool was increased to 13.03 ± 0.13%, a level commensurate with that of non‐inoculated unstressed plants. This increase may be due in part to an increase in [^11^C]‐aspartic acid from 25.81 ± 5.67% in stressed non‐inoculated plants to 38.06 ± 13.70% in inoculated plants (Figure 5C), though we note levels of [^11^C]‐glutamic acid were reduced from 23.72 ± 5.22% in non‐inoculated saline stressed plants to 11.87 ± 4.27% in inoculated stressed plants matching levels of unstressed plants. Furthermore, inoculation was seen to significantly reduce levels of [^11^C]‐raffinose to 0.58 ± 0.15% also matching those levels seen in unstressed plants.

Stress imposed by nitrogen limitation presented a very different profile in new carbon partitioning than salinity stress. Results (Figure 4D) showed significant increases in the soluble sugar pool to 44.08 ± 2.72%, and amino acid pool to 9.05 ± 3.04% relative to 32.62 ± 3.05% and 4.30 ± 0.64% for the same metabolite pools, respectively in non‐inoculated stressed plants. This response was compensated by significant decreases in the acidic metabolite pool to 2.30 ± 0.81% relative to 14.27 ± 1.82% in non‐inoculated unstressed plants. Introduction of the microbial inoculant onto nitrogen stressed plants resulted in an increase in the structural/hydrophobic pool to 32.81 ± 4.21% relative to non‐inoculated stressed plants. The soluble sugar pool was significantly reduced to 39.13 ± 3.25% from that of non‐inoculated stressed plants bringing it in line with the behavior of non‐inoculated unstressed plants. Within this fraction the composition of individual sugars changed only slightly with noted increase in glucose/fructose and raffinose sugars, but sucrose remained the dominant sugar. The amino acid pool was significantly reduced in the presence of inoculant to 1.86 ± 0.34%, below that of non‐inoculated stressed plants, but similar to levels of unstressed plants. Noted compositional changes within this fraction were the significant decreases in serine and glycine amino acids from 25.45 ± 7.74% to 6.96 ± 2.36% and 25.56 ± 4.08% to 12.02 ± 4.08%, respectively, for inoculated versus non‐inoculated plants grown under N limitation (Figure 5D,E). The decreases were compensated by the significant increases in alanine and asparagine from 2.73 ± 1.59% to 10.58 ± 3.59% and 18.73 ± 5.61% to 29.73 ± 3.11%, respectively, for inoculated versus non‐inoculated plants grown under N limitation (Figure 5D,E). Finally, the basic metabolite pool and acidic metabolite pool remained significantly reduced at 14.67 ± 2.11% and 6.24 ± 2.26%, respectively relative to unstressed plants.

### 3.4 Starch analysis

**FIGURE 7:**
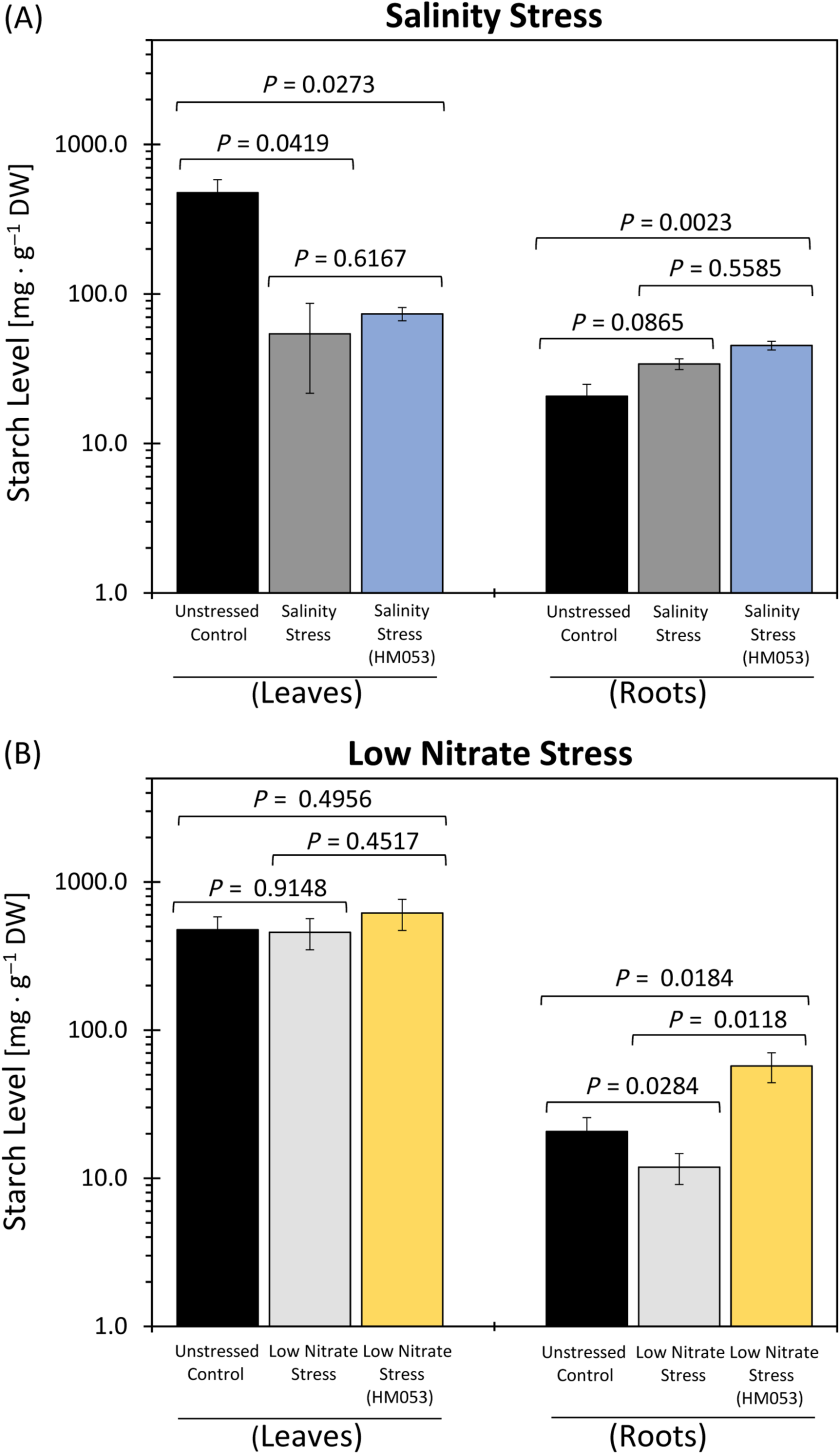
Effect of salinity and low nitrogen stresses on tissue starch. Tissue starch levels are presented for shoots and root tissues as a function of salinity stress (A) and low nitrogen stress (B) and as a function of inoculation with HM053 *Azospirillum brasilense*. Data reflect average values (± se) from *n* = 9–12 biological replicates. Levels of significance are reflected by the included *P*‐values for each figure when comparing the following: (i) non‐inoculated stressed plants to unstressed control plants; (ii) inoculated stressed plants to non‐inoculated stressed plants; and (iii) inoculated stressed plants to unstressed control plants. *P*‐values less than 0.05 were considered statistically significant

The increased root growth trait observed under nitrogen limiting growth in the present study prompted our questioning what carbon resources are being utilized in support of this growth trait. For this reason, shoots and roots of plants were subjected to starch analysis as a function of the imposed growth conditions (Figure 7). Non‐inoculated unstressed plants exhibited levels of starch in leaves and root of 477.0 ± 104.9 and 20.7 ± 5.0 mg g^−1^ dry weight (DW), respectively. Consistent with the literature, root starch levels were always significantly less than leaf starch levels, regardless of the growth conditions (Ning et al., 2018; Y. Wu et al., 2019). Stress from salinity caused a significant reduction in leaf starch levels relative to unstressed plants from 477.0 ± 104.9 to 54.2 ± 32.5 mg g^−1^ DW, and a slight elevation in root starch levels from 20.7 ± 5.0 to 34.0 ± 4.2 mg g^−1^ DW (Figure 7A), though not statistically significant. Introduction of microbial inoculant onto this stress continued to cause suppressed leaf starch levels (73.7 ± 7.5 mg g^−1^ DW) relative to non‐inoculated unstressed plants, while root starch was increased, now significantly (45.2 ± 3.1 mg g^−1^ DW) relative to unstressed plants. Stress from nitrogen limitation (Figure 7B) had no significant impact on leaf starch levels relative to unstressed plants (477.0 ± 104.9 versus 457.3 ± 108.5 mg g^−1^ DW, respectively), but significantly reduced root starch levels from 20.7 ± 5.0 to 11.9 ± 2.8 mg g^−1^ DW, respectively. Interestingly, while introduction of the microbial inoculant onto this stress had no effect on leaf starch levels root starch increased significantly to 57.3 ± 13.1 mg g^−1^ DW levels relative to both non‐inoculated stressed plants and non‐inoculated unstressed plants.

### 3.5 ICP‐MS analysis of plant sodium, potassium, and calcium

**FIGURE 8:**
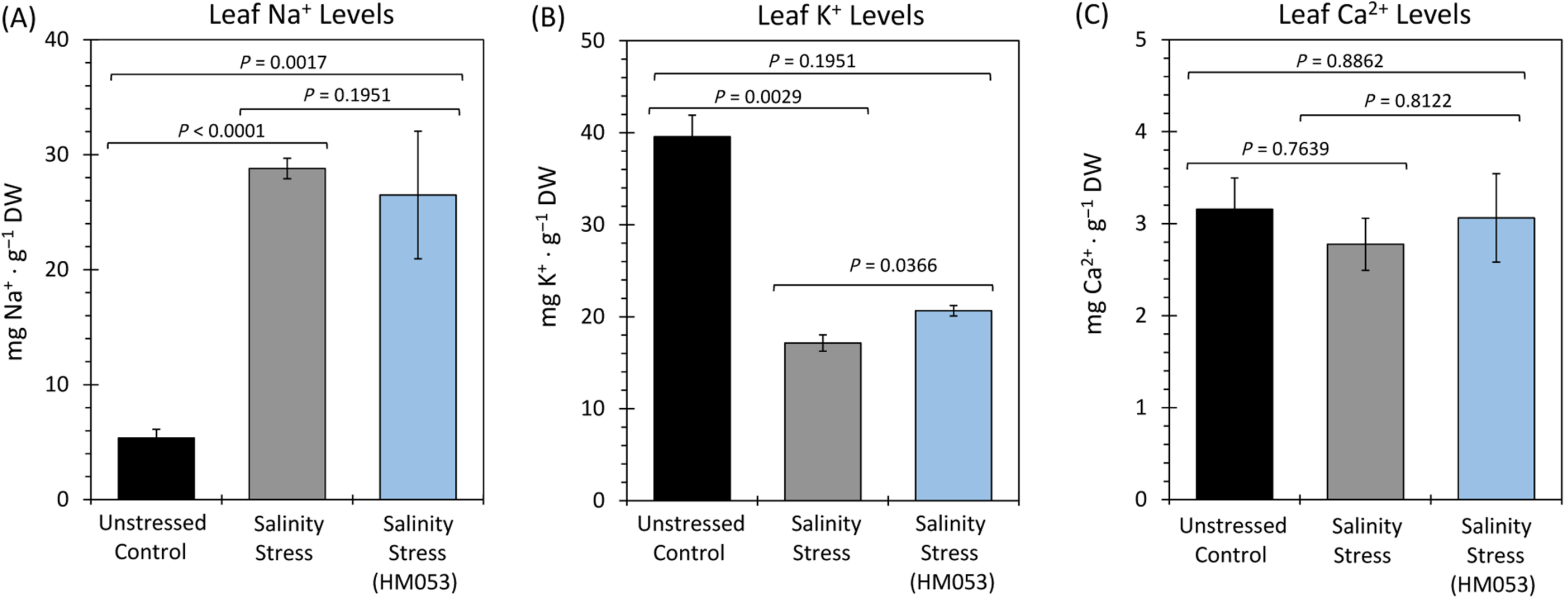
Effect of salinity stress on tissue Na^+^, K^+^ and Ca^2+^ levels. ICP‐MS data show levels of Na^+^ (A), K^+^ (B), and Ca^2+^ (C) in shoot and root tissues of non‐inoculated unstressed control plant, non‐inoculated salt stressed plants and HM053 *Azospirillum brasilense* inoculated salt stressed plants. Data reflect averages (± se) from *n* = 6–9 biological replicates. Levels of significance are reflected by the included *P*‐values for each figure when comparing the following: (i) non‐inoculated stressed plants to unstressed control plants; (ii) inoculated stressed plants to non‐inoculated stressed plants; and (iii) inoculated stressed plants to unstressed control plants. *P*‐values less than 0.05 were considered statistically significant

We examined whether HM053 *A. brasilense* influenced levels of sodium ions as well as other metal ions within the host that are important in regulating salinity stress. Potassium, a potent osmolyte (Nahar et al., 2016), was examined along with calcium—the latter being implicated in signaling processes that trigger plant adaptive responses to salinity (Seifikalhor et al., 2019). Results in Figure 8A showed that leaves typically possessed 5.5 ± 0.9 mg gDW^−1^ of Na^+^ in tissues of unstressed control plants which became significantly elevated to 29.2 ± 1.1 mg gDW^−1^ during salt stress. Introduction of the microbial inoculant to this stress did not alter the elevated Na^+^ levels in foliar tissues which remained at 26.5 ± 5.0 mg gDW^−1^. Furthermore, leaf K^+^ levels of unstressed control plants were 39.9 ± 2.8 mg gDW^−1^ (Figure 8B) but were significantly reduced to 17.0 ± 0.8 mg gDW^−1^ when plants were subjected to salt stress. While the levels of K^+^ seen in the present work for unstressed and salt stressed plants were 1.6‐to‐2.3 times higher than prior published work (Turan et al., 2010) which may be due to differences in the way plants were grown, the systematic trend showing a reduction in K^+^ levels with salt stress was the same. Introduction of microbial inoculant to salinity stress caused a slight, but significant increase in leaf K^+^ to 20.9 ± 0.6 mg gDW^−1^ though this was still significantly below the K^+^ level observed for the unstressed plant. Finally, leaf Ca^2+^ levels remained constant at approximately 3.15 ± 0.4 mg gDW^−1^ (Figure 8C) for unstressed, salt stressed, and inoculated salt stressed plants.

## 4 DISCUSSION

### 4.1 Relief of salinity stress symptoms using HM053 *A. brasilense*

Excessive salt stress can restrict plant growth due to induced ionic, osmotic, or even oxidative stresses tied to the high levels of sodium ions taken up by the plant (Ilangumaran & Smith, 2017; Munns & Tester, 2008; Negrão et al., 2017). Since a plant's root system is the major organ responsible for uptake of water and nutrients from the soil, understanding their function becomes crucial for enhancing plant resistance to salt stress as they are the gateway to regulating Na^+^ acquisition and translocation to the foliar tissues (Jung & McCouch, 2013). Typically, in the presence of high concentrations of Na^+^ ions, plants will experience a decline in primary root growth due to inhibition of cell division and elongation of root epidermal cells (Jung & McCouch, 2013; Rahnama et al., 2011) with compensatory growth stimulation of lateral roots. Fine root systems are better equipped to allow plants to track non‐saline areas of the soil for water, minerals, or other needed resources until exploitation of heavy salt concentrated areas become unavoidable (Alqarawi et al., 2014; Campanelli et al., 2013; Q. S. Wu et al., 2010).

In the present work, similar root growth traits were observed where saline stressed plants exhibited shorter roots and stimulated growth of fine roots resulting in an increase in total root biomass relative to unstressed plants. Although no change in plant carbon input via ^11^CO_2_ fixation was observed with this stress, nor a change in export of leaf ^11^C‐photosynthates, a higher portion of the ^11^C‐photosynthates that exported from the load leaf preferentially allocated to the roots, presumably, in support of enhanced growth in that tissue. This would suggest less carbon was available to support stem growth. Although this feature was not examined in the present work, it was noted that starch levels in salt stressed roots were significantly higher than in unstressed roots, while starch levels in salt stressed shoots were significantly lower than in non‐stressed shoots that could be attributable to the observed changes in carbon allocation.

Salinity stress was shown to alter plant central metabolism. As noted, new carbon partitioning into glutamic acid, aspartic acid, and asparagine was increased under this stress. These three amino acids are intimately tied by their biosynthetic pathways, with asparagine being an end‐product. As a proteinogenic amino acid, asparagine is a structural component of proteins, but its role in plant biology extends beyond this (Oddy et al., 2020). Asparagine has an essential role in nitrogen storage and transport, acting as the main transport molecule of reduced nitrogen in the vasculature, likely because it is the amino acid with the highest nitrogen to carbon ratio (Gaufichon et al., 2010; Lea et al., 2007). While asparagine plays an important role in plant development, it has been shown to accumulate in response to many stressors including salinity (Lea et al., 2007). Asparagine's role here may involve both nitrogen remobilization as well as cellular ammonia detoxification. High salinity can induce a general energy stress within the cell due to ROS bursts that causes proteolysis (Hildebrandt et al., 2015), a process that results in a high cellular ammonia‐to‐hexose ratio (Beato et al., 2014; Lastdrager et al., 2014). However, plant cells have adapted ways to deal with excess ammonia preventing toxic build‐up by recycling it into amino acids and particularly mobile asparagine leveraging the GS/GOGAT cycle and asparagine synthetase. In addition to the above roles, it has also been suggested that asparagine may act as an osmolyte during salt stress (Rashmi et al., 2019).

Besides changes in essential amino acids, we observed that salinity stress caused a significant rise in “new” carbon partitioning in raffinose. Besides being a storage and transport form of carbohydrate, raffinose can often play a role in abiotic stress tolerance in higher plants. Like many of the non‐reducing sugars, raffinose can function as a protectant preventing protein denaturation (Paul et al., 2008). We suspect that the repartitioning of “new” carbon into raffinose at the expense of sucrose may have been an attempt by the plant to build some protection against the imposed salinity stress.

Several past works have shown that the presence of certain microorganisms (Cappellari & Banchio, 2020) and arbuscular mycorrhizal fungi (Evelin et al., 2019; Kapoor et al., 2008) can mitigate the effects of salinity stress. In the present work, introduction of HM053 *A. brasilense* during maize growth under salinity stress appeared to re‐instate a root architecture much like unstressed plants—that is, longer roots were apparent with less fine root structure—suggesting some degree of relief on the host's stressed state. ICP‐MS data suggested that a mechanism for microorganism induced tolerance to salt was not due to suppression of Na^+^ uptake by the host. Indeed, foliar Na^+^ levels remained elevated regardless of inoculation. While inoculation did increase foliar K^+^ levels over that of non‐inoculated stressed plants which was significant, this new level of K^+^ was still well below that of unstressed control plants. Hence, we do not believe the relief seen of the salt stress symptoms was due to a sufficient increase in K^+^ acting as an osmolyte. Our ICP‐MS analyses also examined foliar Ca^2+^ content since plant response to hyperosmotic tension caused by high levels of Na^+^ can be closely linked to Ca^2+^ channels and Ca^2+^ sensing (Seifikalhor et al., 2019). However, we saw no change in Ca^2+^ levels between unstressed controls, salt stressed plants and salt stressed plants with HM053 inoculation. It is acknowledged that such measurements say little about whether cellular Ca^2+^ signaling was altered as such measurements were beyond the scope of this paper.

One mode of positive action on the part of the inoculant was its influence on host carbohydrate metabolism. It was noted that inoculation significantly increased the partitioning of “new” carbon into the soluble sugar pool at the expense of amino acid synthesis and other metabolite biosynthesis. Such alteration in metabolic sugar regulation corresponded well with the observed increased transport of ^11^C‐photosynthates at the whole‐plant level, and with the increased exudation of non‐acidic substrates from the roots. Many sugars will behave as potent osmolytes aiding in building cellular tolerance to hyperosmotic tension caused by high levels of Na^+^ (Nahar et al., 2016). Hence, it is suspected this is the primary mechanism of action for microbial relief of salinity stress.

### 4.2 Relief of nitrogen deficiency symptoms using HM053 *A. brasilense*

The heterogeneous distribution of nitrogen in the soil has forced plants to adapt by altering their root architecture to enhance nutrient acquisition (López‐Bucio et al., 2003). This plasticity of roots under non‐ideal conditions enables them to better forage for resources when experiencing deficiencies of nitrogen, or other essential nutrients (Callahan et al., 1997). Past studies have shown that the influences of nitrogen limitation on maize root growth can be very dependent on variety, but for the most part, roots were longer when subjected to this stress (Kalauni et al., 2016). Consistent with this trend, the present study showed evidence of increased root length when plants were subjected to nitrogen limitation. The carbon source supporting this root growth was of particular interest. Results from the carbon‐11 studies revealed that ^11^CO_2_ fixation was reduced by 40% under nitrogen limiting conditions as were leaf export and root allocation of [^11^C]‐photosynthates. Hence, less “new” carbon was available in support of root growth. It was noted, however, that root starch levels were significantly reduced in roots of nitrogen stressed plants relative to non‐inoculated unstressed plants. This observation suggests that less carbon was placed into storage pools but rather was partitioned into structural components in support of cell wall development. Interestingly, introduction of the microbial inoculant onto the nitrogen limiting stress bolstered root starch levels surpassing that in unstressed plants. This might be a reasonable expectation since a normal root growth trait was re‐instated like that of the unstressed control plants.

The other important observation to note here is the loss of red pigmentation in roots of HM053 exposed plants grown under nitrogen limitation. The presence of anthocyanins is usually associated with red pigmentation of plant tissues. In past work involving Arabidopsis, nitrogen deficiency consistently induced transcript levels of key genes involved in anthocyanin biosynthesis, including *F3′H*, *DFR*, *LDOX*, and *UF3GT* causing systemic accumulation of the metabolite (Y. Zhang et al., 2017). Likewise, the present study showed extensive red pigmentation of roots of plants grown under nitrogen limitation and when critically examined showed a significant elevation in root anthocyanin levels. Part of this action can be attributed to increased partitioning of new carbon (as ^11^C) into amino acids, noting that anthocyanins are products of phenylalanine and tyrosine which metabolize to cinnamic acid and coumaric acid, respectively in the phenyl propanoid pathway. Anthocyanin biosynthesis is also induced by sucrose (Loreti et al., 2008; Teng et al., 2005). In this work, nitrogen limited growth caused a significant increase in “new” carbon (as ^11^C) partitioning into soluble sugars with sucrose dominating that pool. This change in carbon metabolism could be the reason for the increased root anthocyanin levels observed under nitrogen limiting growth. Furthermore, the introduction of the microbial inoculant onto this stress with the resultant reversal in the sugar pool back to a level commensurate with non‐inoculated unstressed plants could account for the loss of root pigmentation attributable to the observed suppression of anthocyanin.

As a diazotroph, HM053 *A. brasilense* has been implicated with relieving nitrogen stress in the *Setaria viridis* grass by supplying extraneous nitrogen to its host via biological nitrogen fixation (Pankievicz et al., 2015). Whether or not a similar mechanism of action is in play for the maize system examined in the present work remains to be seen. Future studies using radioactive ^13^NN to directly quantify host nitrogen uptake via biological nitrogen fixation will be needed (Pankievicz et al., 2015). Alternatively, this microbial inoculant could upregulate host nitrate uptake under nitrogen limiting growth conditions. Although less nitrogen is available in the growth medium, the increase in nitrate uptake kinetics might compensate for the deficiency. This too can be examined in future studies using the radioactive ^13^NO_3_^−^ tracer. Indeed, past studies in birch saplings using ^13^NO_3_^−^ showed an alteration in the tracer uptake kinetics with nitrate availability (Ferrieri et al., 2018).

## 5 CONCLUSIONS

This work showcases the power and utility of using carbon‐11 radiotracing to map changes in whole‐plant physiology and “new” carbon metabolism giving insight into how PGPB can interact and influence the state of their host plant. Clearly, the present work shows evidence that HM053 *A. brasilense* can act through many diverse mechanisms of action relieving plant abiotic stress symptoms caused by salinity and nitrogen limitation. However, we acknowledge that the results reported in the present studies only reflect the effects over a short window of the plant's life cycle. Questions that remain unanswered include whether the inoculants administered would continue to flourish through the entire growing season, and whether the short‐term beneficial effects seen in the present work can be sustained through the season resulting in crop yield improvement.

## CONFLICT OF INTEREST

The authors declare that they have no competing interests.

## AUTHOR CONTRIBUTIONS

*Experimental design and supervision*: Richard A. Ferrieri. *Data collection and analysis*: Avery Powell, Stacey L. Wilder, Alexandra B. Housh, Garren Powell, Stephanie Scott, Spenser Waller, Mary Benoit, Michael J. Schueller, and James M. Guthrie. *Initial draft*: Richard A. Ferrieri and Stacey L. Wilder. *Final draft*: All authors.

## Supporting information


**Figure S1** Plant growth.

**Figure S2** Schematic of radiotracer workflow.

**Figure S3** Microbial drop plate assay.

## Data Availability

All data needed to evaluate the conclusions in the paper are present in the main text. Data will be made available upon request to corresponding author.
