# Supplementary figures and images for: Examining effects of rhizobacteria in relieving abiotic crop stresses using carbon‐11 radiotracing
Source: Physiol Plant. 2022 Mar 29;174(2):e13675. doi: 10.1111/ppl.13675 (PMC9310733; doi:10.1111/ppl.13675)

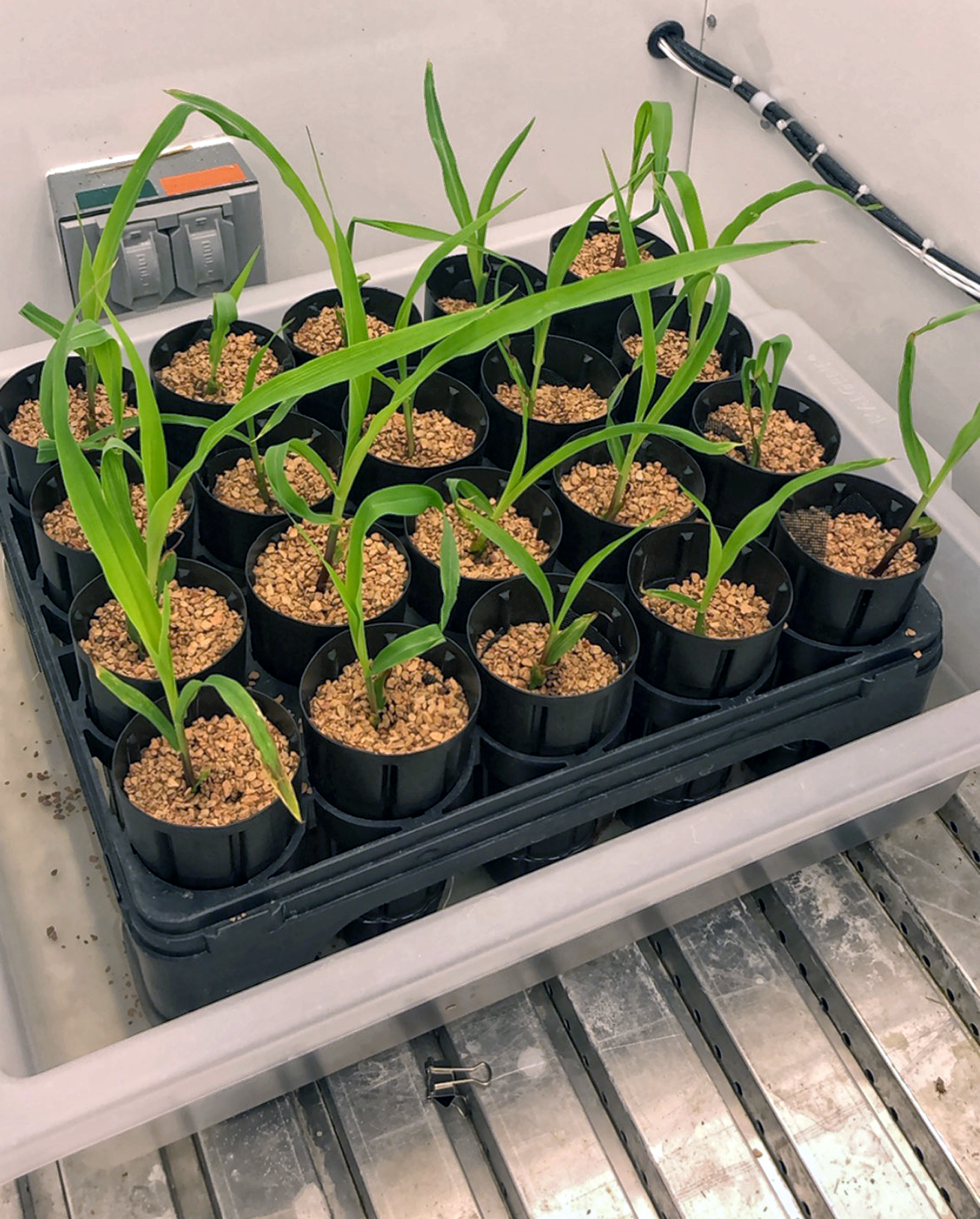

Supplement: Supplementary file 1 — Figure S1 Plant growth. [file PPL-174-0-s001.tif]

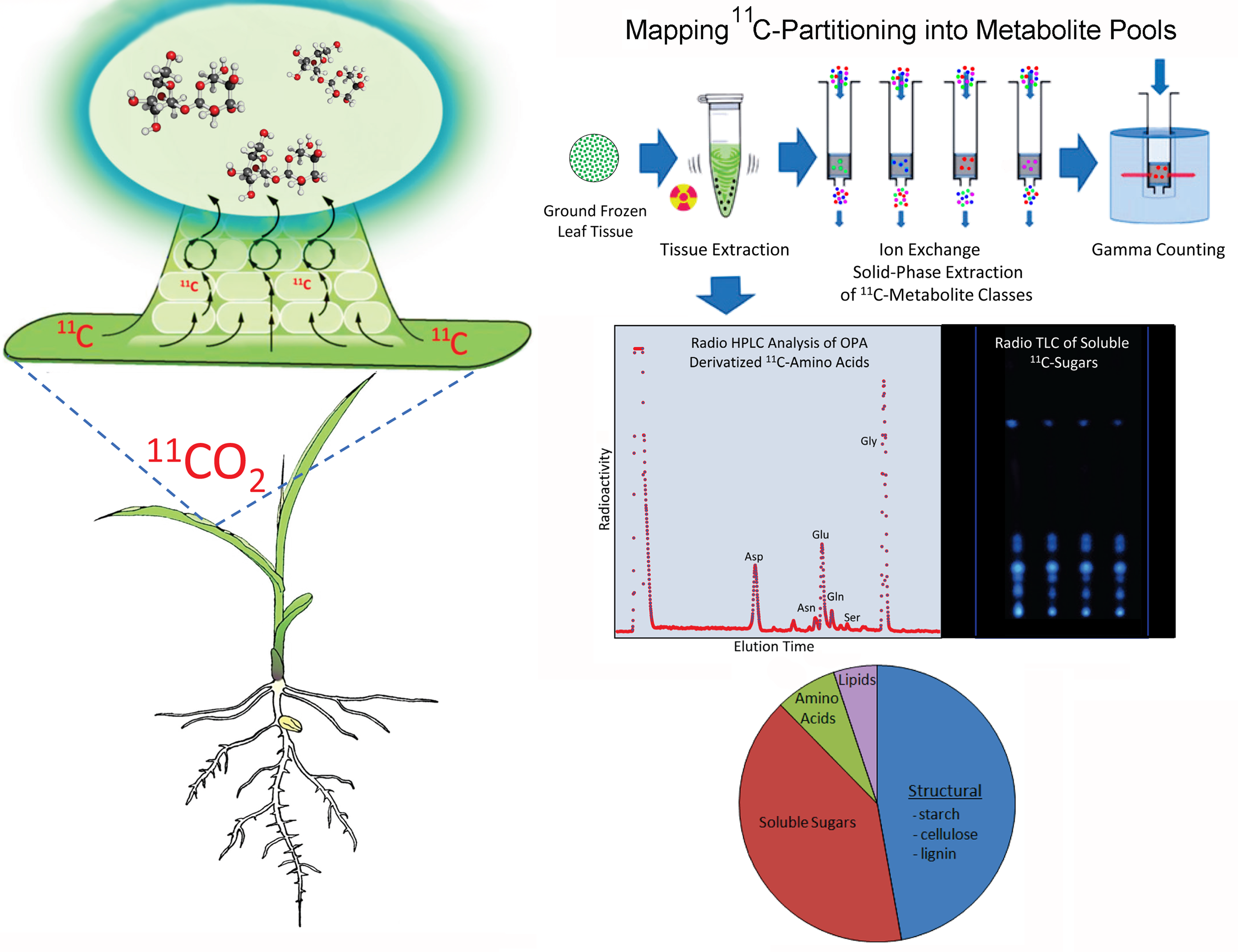

Supplement: Supplementary file 2 — Figure S2 Schematic of radiotracer workflow. [file PPL-174-0-s003.tif]

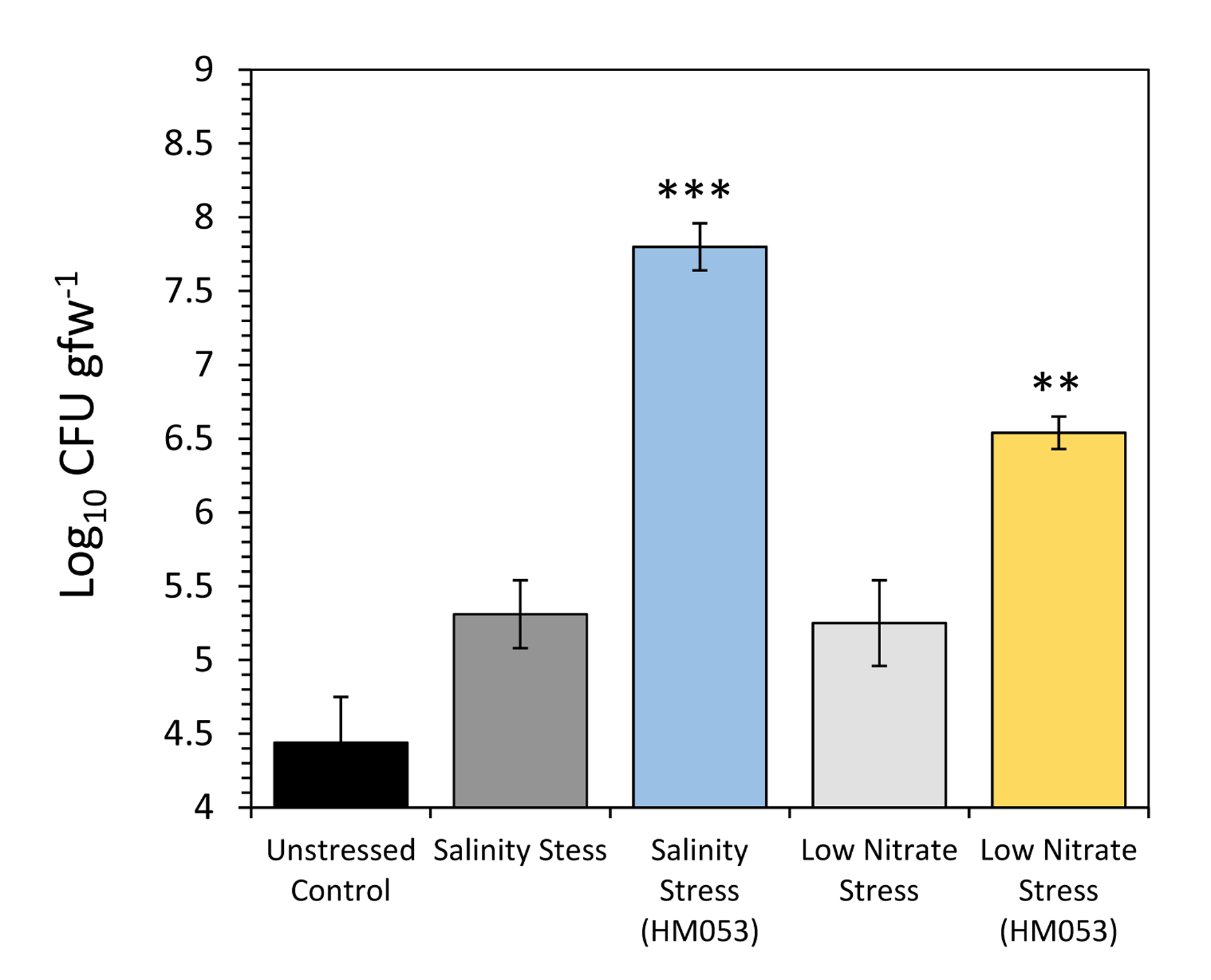

Supplement: Supplementary file 3 — Figure S3 Microbial drop plate assay. [file PPL-174-0-s002.tif]
